# Unraveling the full impact of SPD_0739: a key effector in *S. pneumoniae* iron homeostasis

**DOI:** 10.1128/spectrum.01331-24

**Published:** 2024-10-29

**Authors:** Edroyal Womack, Melina Antone, Zehava Eichenbaum

**Affiliations:** 1 Department of Biology, Georgia State University, Atlanta, Georgia, USA; The Ohio State University College of Dentistry, Columbus, Ohio, USA

**Keywords:** *Streptococcus pneumoniae*, pneumococcus, heme transport, iron acquisition, iron transport, iron metabolism, nucleoside scavenging, surface proteins, lipoproteins

## Abstract

*Streptococcus pneumoniae* is a common member of the nasopharynx commensal microflora and the leading etiological agent of bacterial pneumonia in young children and aging adults. SPD_0739, a highly expressed lipoprotein, is the predicted substrate-binding component of an ABC transporter linked to the uptake of nucleosides and heme by independent studies (named PnrA or Spbhp-37, respectively). Here, we demonstrate that SPD_0739 binds heme *in vitro* and contributes to the bacterial binding to hemoglobin. A ∆*spd_0739* strain exhibited growth attenuation that was relieved by the inactivation of the *piuBCDA* transporter. Knocking out *spd_0739* in the wild type, or the Δ*piuBCDA* strain resulted in heme accumulation, higher sensitivity to heme toxicity, and a small growth reduction compared to medium supplemented with a nucleoside mixture. In addition, *spd_0739* loss results in higher iron- and heme-related gene expression and lower H_2_O_2_ production. Altogether, the data are consistent with a role in nucleoside import and show that SPD_0739 does not import heme. Instead, it indirectly influences iron and heme metabolism, linking nucleosides and iron status in *S. pneumonia*e.

**IMPORTANCE:**

*S. pneumoniae* obtains growth essential iron from hemoglobin and other host hemoproteins. Still, the bacterial mechanisms involved are only partially understood, and there are inconsistent reports regarding the function of several transporters implicated in iron uptake. In this study, we clarified the role of PnrA/Spbhp-37, a ligand-binding protein previously linked to nucleoside or heme by different studies. We present data supporting a role in nucleoside scavenging rather than heme import and reveal that PnrA/Spbhp-37 modulates iron and heme uptake, likely by influencing the nucleoside cellular pool. Hence, this work provides a new understanding of a process critical to the pathophysiology of a significant human pathogen. Moreover, PnrA/Spbhp-37 is an abundant and immunogenic surface protein that is highly conserved. Hence, this study also clarifies the function of a promising vaccine target.

## INTRODUCTION

The human body contains 3–5 g of iron; however, iron is not found free; instead, it is sequestered by proteins to avoid toxicity and defend from infection by iron-requiring pathogens. Heme holds ~70% of the bodily iron, most in hemoglobin, and the rest in other hemoproteins such as myoglobin and heme-containing cytochromes. Non-heme iron serves as a cofactor for iron enzymes (often in Fe–S clusters), and the spare metal is insulated intracellularly in the storage protein, ferritin, or found in hemosiderin complexes. Various carrier proteins for ferric iron, heme, and hemoglobin (e.g., transferrin and hemopexin) restrict free iron and heme levels in the extracellular compartment (1). During infection, the host reduces iron absorption and metal levels in circulation and at infection sites as part of the innate response. Altogether, the host’s iron-withholding mechanisms result in an extremely low concentration of free iron far below the concentration required for bacterial growth (2–4).

*Streptococcus pneumoniae* is an obligate human pathogen that colonizes the human nasal pharynx early in childhood and persists asymptomatically. Colonizing pneumococci can shift into a virulent state and cause a disease spectrum that ranges from mild to life-threatening conditions, including otitis media infections, bacterial pneumonia, bacteremia, and meningitis (5). Pneumococcal burden is the highest in adults over 50 years and children younger than 5 years of age, with more than 800,000 deaths of children reported annually worldwide. The World Health Organization predicts that with the rise in the aging population, hospitalization for pneumococcal diseases will increase by 96% and will cost ~$5 billion per year by 2040 (6). Current immunization programs are based on conjugated vaccines that cover prevalent pneumococcal capsule types, though these vaccines protect against a fraction of the pneumococcal serotypes. Immunization has led to an increase in human carriage of non-vaccine serotypes, and infections by vaccine-escape isolates have been reported (7).

The opportunistic pathogen *S. pneumoniae* needs iron for growth and can obtain the metal from heme and hemoglobin. In addition to providing essential iron, hemoglobin also promotes pneumococcal adaptation to the host mucosal surface, planktonic growth, and biofilm development (8–11). We recently discovered that the hydrogen peroxide (H_2_O_2_) produced and secreted by pneumococcus during growth in an aerobic environment degrades the heme moiety of hemoglobin, liberating the iron in the extracellular compartment (12, 13). This unique mechanism offers pneumococci an efficient way to take advantage of the large heme and hemoglobin pool during colonization of the nasopharynx and infection in sites with high oxygen tension. *S. pneumoniae* also removes the heme from hemoglobin and imports and degrades it intracellularly by H_2_O_2_-independent mechanisms yet to be described. This ability likely benefits the pathogen during systemic spread and in niches with low oxygen tension, where H_2_O_2_ production is expected to be limited (14, 15).

Gram-positive pathogens often use heme relay mechanisms consisting of surface-exposed proteins that remove heme from the host hemoproteins and deliver it across the peptidoglycan layers to the substate-binding protein of a membrane-embedded ABC transporter. Shr and Shp of *S. pyogenes* (16–19), and the IsdA, IsdB, IsdC, and IsdH proteins of *Staphylococcus aureus* (20, 21) are well-characterized examples of surface heme relay systems (22–24). To date, such a heme relay mechanism has not been described in pneumococcus, but a few ABC transporters have been linked to importing heme across the cytoplasmic membrane (11, 25–28).

Romero-Espejel et al. identified a 37-kD lipoprotein predicted to be the substrate-binding protein of an ABC transporter from the *S. pneumoniae* R6 strain in a preliminary screen for proteins that bind heme and hemoglobin and named it Spbhp-37 (NCBI GeneInfo Identifier, gi|15900732 or SPR 0747 in the Kyoto Encyclopedia of Genes and Genomes (KEGG) (10, 29). A subsequent study demonstrated that Spbhp-37 binds hemoglobin in high affinity and that adding Spbhp-37 antiserum to the culture interferes with pneumococcal growth on hemoglobin as an iron source (30). Following these reports, we examined the phenotype of a knockout mutant of *spbhp-37* ortholog in the D39 strain (*spd_0739*) (9). We showed the mutant growth is attenuated in the rich laboratory THYB and that this phenotype is aggravated when the bacteria are cultivated in iron-depleted THYB supplemented with hemoglobin as the single source of iron. Moreover, the D39 ∆*spbhp-37* strain lost the ability to grow in iron-depleted THYB containing free heme (9). Other *in silico* and functional studies, however, suggest *spbhp-37* in D39, and its ortholog in TIGR4 (*sp_0845*), encodes the ligand-binding component of a nucleoside ABC transporter and designates it as PnrA (pneumococcal nucleoside receptor A) (31, 32). These investigations showed that knockout mutants of D39 *spd_0739* and TIGR4 *sp_0845* demonstrated reduced uptake of guanosine and increased resistance to the toxic nucleoside analog, 5FU (33). Recently, the structure of the TIGR4 protein (SP_0845) bound to adenosine, guanosine, cytidine, thymidine, or uridine was resolved, and additional biochemical characterization revealed that the protein exhibits a preference for purine ribonucleosides (31). In summary, separate studies linked the same lipoprotein to the uptake of heme or nucleosides, calling it Spbhp-37 or PnrA, respectively.

The *spbhp-37*/*pnrA* gene belongs to the pneumococcal core genome and encodes a highly conserved immunogenic protein that serves as a plentiful component of the pneumococcal cell envelope (34). Here, we further investigate the function of Spbhp-37/PnrA protein in pneumococcal biology. We confirmed that the D39 Spbhp-37/PnrA protein binds heme *in vitro* and showed that it promotes hemoglobin binding to the pneumococcal surface. We found that this protein does not contribute to heme uptake; instead, it influences the cellular heme content by indirectly inhibiting the expression of iron and heme uptake genes and activating H_2_O_2_ production. Hence, the data identify Spbhp-37/PnrA as a new mediator that links nucleoside uptake to H_2_O_2_ production and iron metabolism in *S. pneumoniae*. To avoid confusion, we refer below to *spbhp-37*/*pnrA* using the gene name in the D39 genome in KEGG, *spd_0739* (29, 35).

## RESULTS

### SPD_0739 protein binds heme *in vitro* and contributes to hemoglobin binding to the pneumococcal cell surface

**Fig 1:**
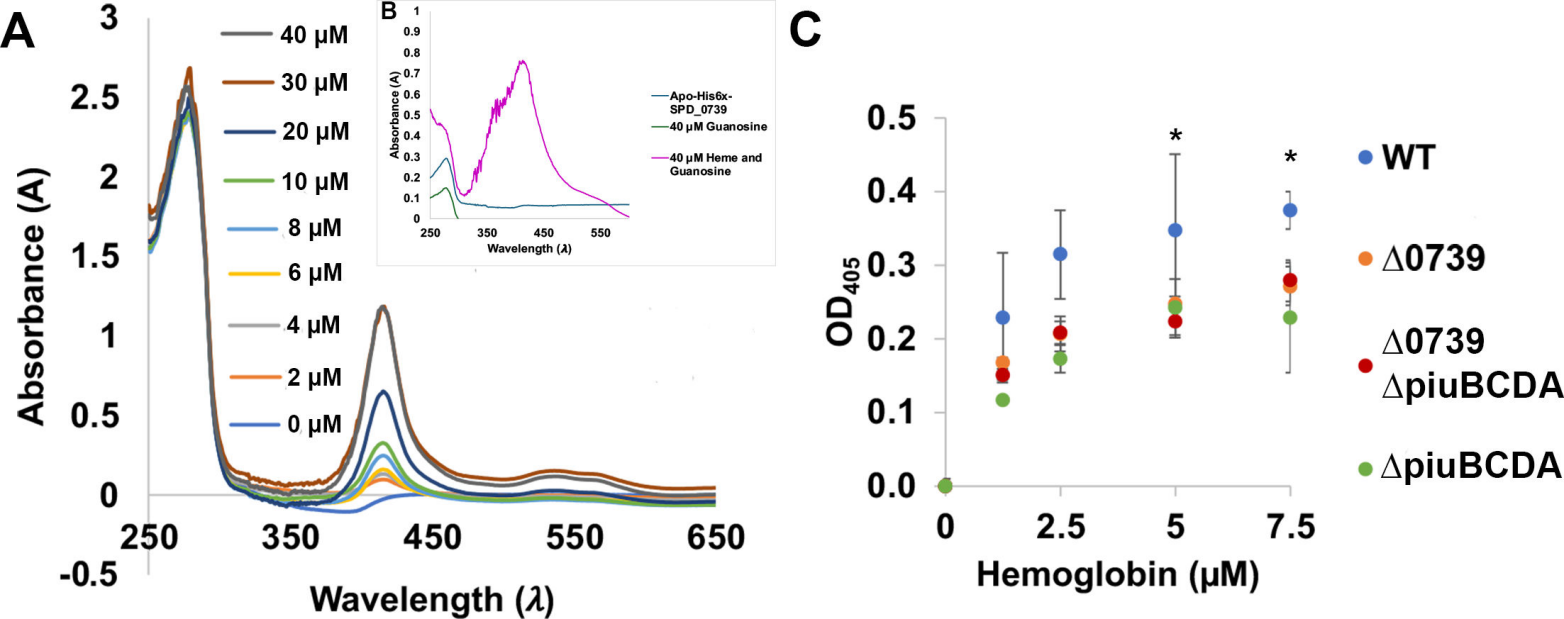
SPD_0739 protein binds heme *in vitro* and contributes to hemoglobin binding to pneumococcal cell surface. **(A**) Heme binding by purified SPD_0739 protein. Purified His_6_-tagged SPD_0739 (10 µM) was allowed to interact with increasing increments of heme. The reaction absorbance (250–650 nm) was monitored after a 5-min incubation. The buffer with equimolar concentrations of heme was used as a blank. The growing Soret peak (416 nm) indicates SPD_0739 binds heme. The inset shows a protein marker next to a purified His_6_-SPD_0739. (**B**) Heme binding by His_6_-SPD_0739 in the presence of guanosine. The same as in (A), only that SPD_0739 was incubated with 40 µM guanosine prior to incubation with 40 µM heme. (**C**) Hemoglobin binding to pneumococcal cell surface. Microtiter plates were coated with *S. pneumoniae* overnight cultures (OD_600_ 1.0), washed, and allowed to interact with increasing concentrations of hemoglobin. Hemoglobin binding was detected using rabbit hemoglobin antiserum and anti-rabbit antibody conjugated to alkaline phosphates. The values obtained in wells with untreated cells (no hemoglobin, negative control) were subtracted from the values obtained in wells containing cells treated with hemoglobin. The shown data were derived from experiments done in duplicates and repeated at least two times. Statistical significance was determined for each concentration by one-way analysis of variance (ANOVA) with Tukey *post hoc*, where * indicates a *P* ≤ 0.05. Error bars indicate standard deviation.

SPD_0739 ortholog in the R6 strain, SPR_0747, was originally recovered from the pneumococcal lysate by binding to a hemin affinity column. A subsequent surface plasmon resonance (SPR) study showed that the purified SPR_0747 binds hemoglobin in high affinity (10, 30). Consistent with these observations, we previously showed that the ∆*spd_0739* strain is significantly attenuated when cultivated on hemoglobin or heme as the only iron source (9). To further test the hypothesis, we examined SPD_0739 direct interactions with heme. A His_6_-SPD_0739 recombinant protein was expressed and purified from *Escherichia coli* (Fig. 1A). UV-visible spectroscopic inspection revealed the recombinant protein absorbed only at 280 nm, indicating it was purified in the apo form. Incubating SPD_0739 with increasing heme concentration, however, resulted in the gradual formation of a second absorption peak at 416 nm (i.e., a Soret band), which indicates heme binding (Fig. 1A). Since previous growth and uptake studies have linked SPD_0739 to nucleoside import, and structural and biochemical investigations of SPD_0739 homolog in TIGR4 (SP_0845) established it binds nucleosides with a preference for purines (31), we tested if the presence of nucleosides influences SPD_0739 interactions with heme. We preincubated His_6_-SPD_0739 with guanosine (in a 1:4 protein to nucleoside ratio) before incubating and testing for heme binding spectroscopically. We also co-incubated SPD-0739 with guanosine and heme at the same time. In both types of experiments, SPD_0739 exhibited a Soret peak upon incubation with heme (Fig. 1B and data not shown), suggesting guanosine does not prevent heme binding to SPD_0739.

Next, we tested SPD_0739 contribution to pneumococcal interactions with hemoglobin using an enzyme-linked immunosorbent assay (ELISA). We compared hemoglobin binding by immobilized cells of the D39 wild type and isogenic mutants in *spd_0739* and/or the *piuBCDA* importer whose ligand-binding protein, PiuA, was also reported to bind hemoglobin and heme (11). Overnight cultures of D39 wild type, *∆spd_0739*, *∆piuBCDA*, and *∆spd_0739∆piuBCDA* double mutant were used to coat a 96-well microtiter plate (11, 25, 36–38). The pneumococci were allowed to interact with increasing concentrations of hemoglobin, and binding was detected by anti-hemoglobin antibodies (Fig. 1C). We added catalase to the medium to prevent heme degradation and hemoglobin denaturation, thus preventing it from falling out of solution due to interactions with the endogenously produced H_2_O_2_ (12). Deleting either *spd_0739* or *piuBCDA* genes resulted in a 30%–35% reduction in pneumococcal binding to hemoglobin. These observations support the reports that both PiuA and Spd_0739 proteins bind hemoglobin and suggest that both pathways contribute to the interactions of hemoglobin with the pneumococcal cell surface. Surprisingly, deleting both *spd_0739* and *piuBCDA* did not have an accumulative effect, and the double mutant bound hemoglobin to the same extent as the single mutants. This observation implies that hemoglobin binding by PiuA and SPD_0739 is interconnected or that inactivation of both resulted in the upregulation of another hemoglobin-binding protein.

### SPD_0739 negatively influences heme uptake and sensitizes *S. pneumoniae* to heme toxicity

**Fig 2:**
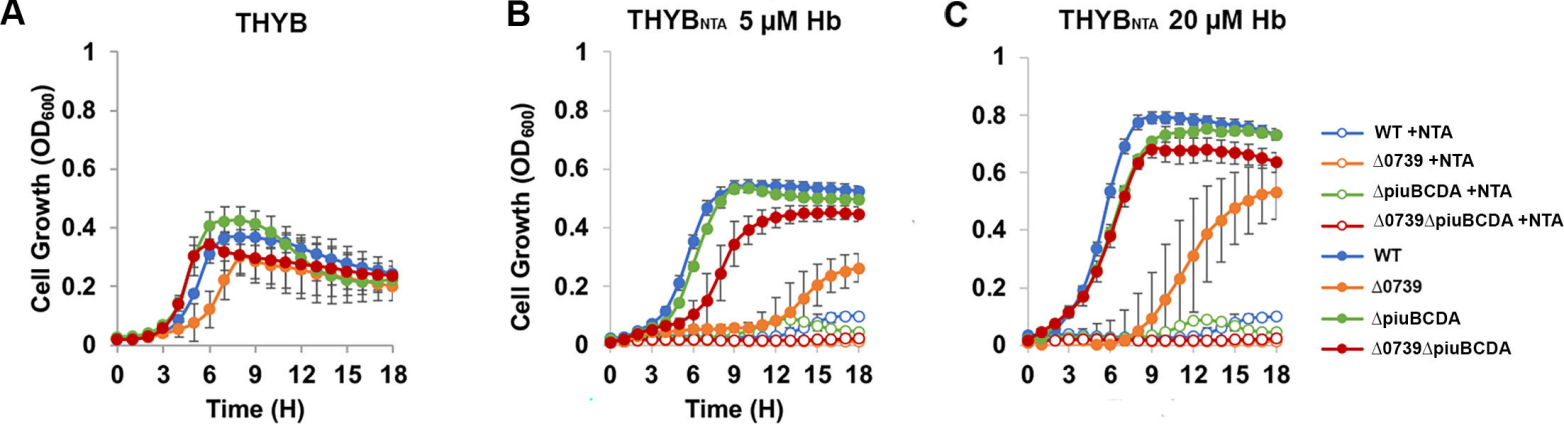
Inactivation of the *piuBCDA transporter* alleviates *∆spd_0739* growth phenotype. *S. pneumoniae* growth in regular THYB (**A**) or THYB containing 3 mM of the iron chelator NTA (THYB_NTA_) with or without 5 µM (**B**) or 20 µM (**C**) hemoglobin. Cells of D39 wild type (WT, blue), ∆*spd_0739* (orange), ∆*piuBCDA* (green), and ∆*spd_0739*∆*piuBCDA* double mutant (red) isogenic strains were used to inoculate fresh media (OD_600_ 0.05). Cultures in 96-well microtiter plates were incubated at 37°C. Growth is expressed in optical density (OD_600_) as measured every hour. Open circles represent cultures grown in the iron-depleted THYB_NTA_, and closed circles represent those cultivated in THYB or THYB_NTA_ with hemoglobin. All experiments were done in triplicates and repeated at least two more times. Each curve shown is derived from an average of three bioreplicates from representative experiments. Error bars indicate standard deviation.

We next tested the use of hemoglobin as an iron source by a single and double mutant in *spd_0739* and *piuBCDA.* The PiuBCDA transporter was first linked to heme import (11, 38) and later shown to recognize Fe^+3^–catachol complexes with much higher affinity (36, 39). Still, we recently demonstrated that *piuBCDA* inactivation leads to a significant reduction in cellular heme content in pneumococci cultivated on hemoglobin as the sole source of iron (12). We hypothesized that the simultaneous loss of both the SPD_0739 and PiuBCDA pathways would exacerbate the phenotype observed with the loss of each transporter, assuming both pathways contribute to cellular heme accumulation. We monitored the growth of the isogenic single and double mutants in THYB (Fig. 2A), THYB_NTA_ (THYB containing 3 mM NTA), and THYB_NTA_ containing 5 or 20 µM hemoglobin (Fig. 2B and C, respectively). NTA chelates the free iron in the medium, and therefore in its presence, the bacteria depend on heme import and intracellular degradation for iron supply. The growth of all strains was inhibited in THYB_NTA_ unless hemoglobin was added to the medium, and all three strains exhibited the best growth in THYB_NTA_ with 20 µM hemoglobin. The deletion of *spd_0739* impaired growth in regular THYB. This phenotype worsened when the mutant was cultured in THYB_NTA_ containing 5 µM hemoglobin, with the mutant exhibiting a 12 h-long lag phase followed by a short logarithmic growth, eventually yielding a very low optical density (Fig. 2B). In THYB_NTA_ containing 20 µM hemoglobin, the ∆*spd_0739* mutant grew better but still exhibited repressed growth compared with the wild-type strain. Surprisingly, the *∆spd_0739∆piuBCDA* double knockout grew much better than the single *spd_0739* mutant in all growth conditions. Hence, inactivating *piuBCDA* not only did not worsen but also relieved most of the growth attenuation caused by the loss of *spd_0739.* Nevertheless, the growth of the double mutant was delayed and slower compared with the wild type and the *∆piuBCDA* strains in THYB_NTA_ with 5 µM hemoglobin. In THYB_NTA_ supplemented with 20 µM hemoglobin, the *∆spd_0739∆piuBCDA* mutant grew similar to the ∆*piuBCDA* strain. Inactivation of *piuBCDA* alone did not significantly impact bacterial growth in THYB or THYB_NTA_ with 5 or 20 µM hemoglobin (Fig. 2). This was expected as we and others have previously noted the absence of a phenotype for the Δ*piuBCDA* strain cultivated on hemoglobin–iron (11, 40).

**Fig 3:**
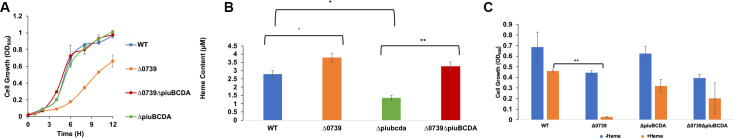
Inactivation of *spd_0739* results in accumulation of cellular heme and increased heme sensitivity. Cultures of D39 wild type (WT, blue), ∆*spd_0739* (orange), ∆*piuBCDA* (green), and ∆*spd_0739*∆*piuBCDA* isogenic strains were allowed to grow in THYB_NTA_ supplemented with 20 µM hemoglobin in 12-well microtiter plates, and growth was monitored. (**A**) *S. pneumoniae* growth over time. (**B**) Heme concentration as determined by the acidified chloroform method in culture samples harvested at the early-log phase (OD_600_ = 0.3) and standardized according to the culture OD_600_. Samples were collected and processed in duplicates from a minimum of two biological replicates. (**C**) Heme sensitivity exhibited by the *S. pneumoniae* isogenic series. Cells were allowed to grow in THYB containing catalase (200 U/µL) with or without 10 µM heme, and the culture OD_600_ was determined after 10 h of incubation at 37°C. A one-way ANOVA with Tukey *post hoc* and Student *t*-test were used to determine statistical significance, where * indicates a *P* ≤ 0.05 and ** ≤0.01. Error bars indicate standard deviation.

We next compared the cellular heme level in the *∆spd_0739*, *∆piuBCDA*, and *∆spd_0739∆piuBCDA* strains. Heme cellular content peaks in the mid-exponential growth phase and then declines (12); hence, we determined heme levels in cells collected at the mid-logarithmic phase of growth (~0.3 OD_600_, Fig. 3A and B). Samples of the isogenic strains grown on hemoglobin iron were collected, and heme content in the cell lysate was determined as described (Fig. 3B). We recover approximately 48% of the amount of heme from the ∆*piuBCDA* strain compared with the wild type, confirming that PiuBCDA supports the cellular accumulation of heme. On the other hand, the loss of *spd_0739* resulted in a 36% increase in heme content compared with the wild type, suggesting *spd_0739* has an inhibitory effect on heme uptake. Inactivating *spd_0739* in the background of ∆*piuBCDA* increased heme content compared with the wild-type strain, proposing that *spd_0739* negatively affects the expression or function of heme uptake mechanisms that are independent of *piuBCDA*. We did not find statistical differences in heme content between the double mutant and *∆spd0739*, but the trend suggests a lower level of heme in the double mutant.

Free heme disrupts membranes and promotes cellular damage by oxidating DNA, proteins, and lipids (41–43). Since the inactivation of *spd_0739* results in heme accumulation, we asked if this mutation also leads to increased heme sensitivity. We tested the growth of the wild type, *∆spd_0739*, *∆piuBCDA*, and the ∆*spd_0739∆piuBCDA* in THYB supplemented with 10 µM heme and catalase. Catalase was included in the medium to prevent heme degradation by the endogenously produced hydrogen peroxide (12, 13). Externally added heme inhibited the growth of all tested strains in THYB. Still, *∆spd_0739* did not grow at all in THYB with 10 µM heme; the wild type, *∆piuBCDA*, and the *∆spd_0739∆piuBCDA* strain were able to grow to a significant extent in this medium exhibiting (~67%, ~51%, and ~52% recovery compared to their growth in THYB, respectfully).

### Inactivation of *spd_0739* leads to the upregulation of iron and heme transport genes and reduces the production of endogenous hydrogen peroxide

**Fig 4:**
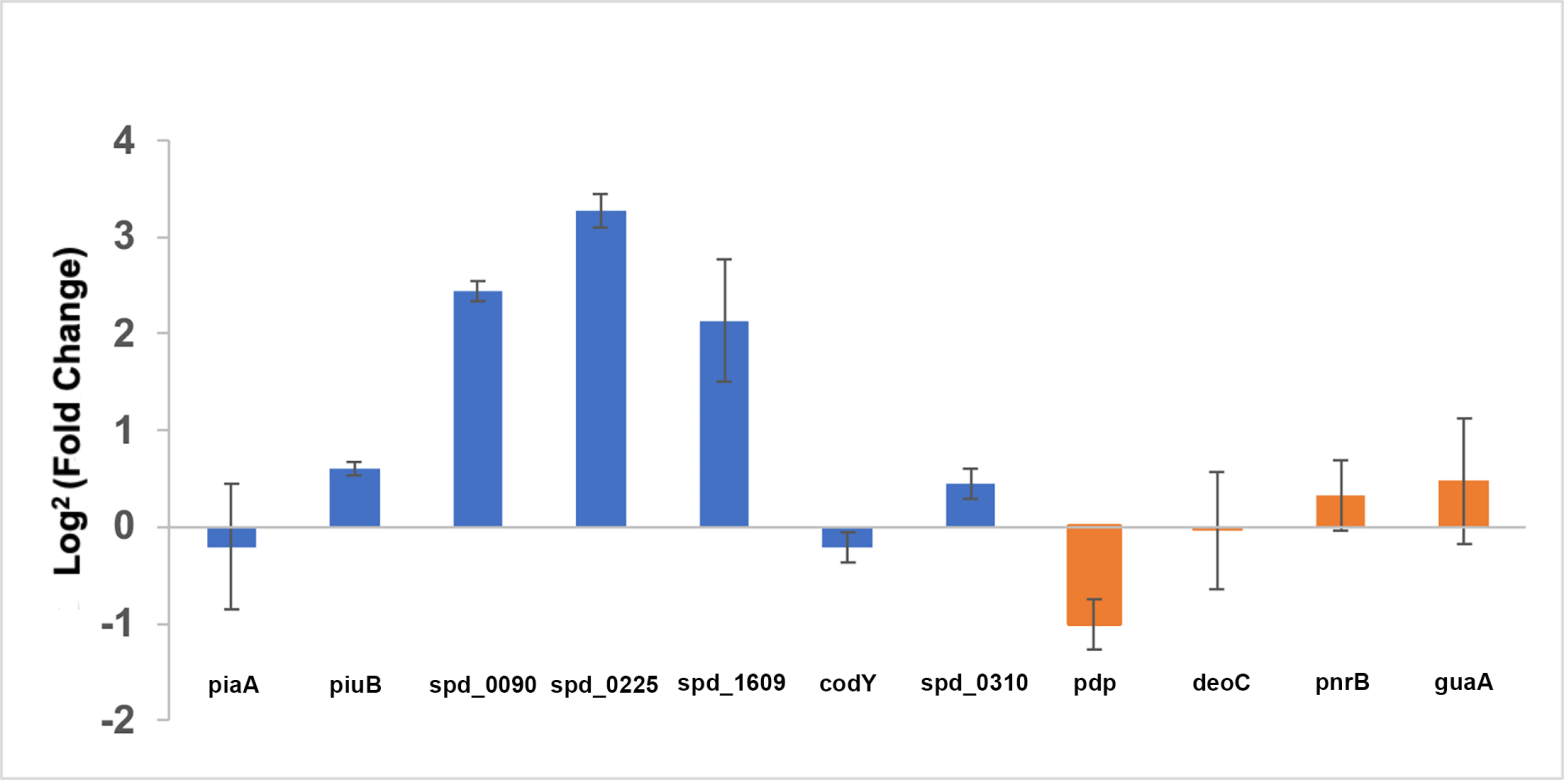
Inactivation of *spd_0739* influences the expression of several genes involved in iron and heme transport and of the pyrimidine nucleoside phosphorylase (*pdp*) gene**.** RNA was prepared from culture samples of the isogenic wild type and ∆*spd_0739* strains grown in THYB up to the mid-log phase. Fold change in gene expression exhibited by the ∆*spd_0739* mutant compared to the isogenic wild-type strain was determined using qRT-PCR. The data were derived from samples collected from two bioreplicates, processed in duplicates.

The observation that the ∆*spd_0739* mutant accumulated more heme compared with the wild-type strain suggests SPD_0739 has a negative influence on heme import. Following up on these observations, we tested how the removal of *spd_0739* affects the expression of iron and heme-related genes. Exponentially growing pneumococci cultivated in THYB were harvested, and the expression of selected genes was analyzed by RT-PCR (Fig. 4A). This study revealed a fivefold upregulation of *spd_0090*, the substrate binding protein of a putative ABC transporter recently implicated in heme import (26). In addition, the ∆*spd_0739* strain exhibited significant elevation in the expression of the ferric uptake genes *pitB (spd_0225)* (25, 40)*,* ~10-fold and *pitA2 (spd_1609)* (44), ~7-fold. We did not see any significant changes in *piuB* expression or *codY*, a nutritional regulator of *piuB*. Similarly, the deletion of *spd_0739* did not impact the ferrochrome-binding protein, *piaA* (45, 46) or *spd_0310,* which is suggested to function as a heme chaperon (47).

Since SPD_0739 is linked to nucleoside scavenging, we also tested how its inactivation affects the expression of genes involved in nucleoside use. Using RT-PCR, we tested the expression of *pdp (spd_0736)*, a pyrimidine nucleoside phosphorylase encoded upstream of *spd_0739* from a separate operon; *deoC (spd_0737)*, a deoxyribose-phosphate aldolase; *pnrB* (*spd_0740)*, the permease of the PnrABC transporter (that is likely expressed independently of *spd_0739*/*pnrA gene)*; and *guaA* (*spd_1274*), a GMP synthase expressed in on a different chromosomal region. We found that the loss of *spd_0739* was associated with a twofold downregulation of *pdp* (Fig. 4).

**Fig 5:**
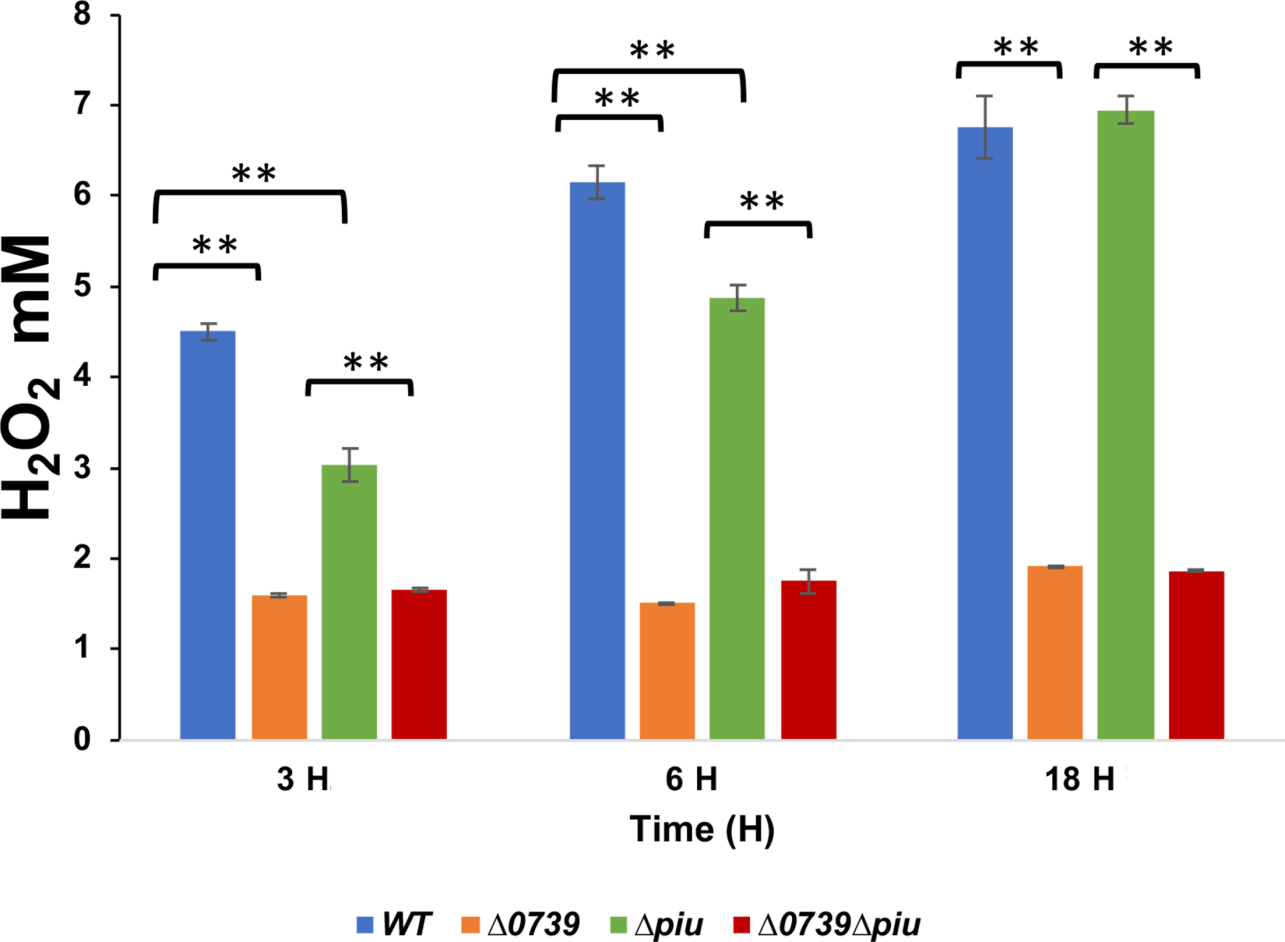
*spd_0739* loss is associated with reduced hydrogen peroxide production. Shown are hydrogen peroxide concentrations found in the culture supernatant of D39 WT, *∆spd_0739*, *∆piuBCDA*, and *∆spd_0739∆piuBCDA* strains grown in THYB. A one-way ANOVA with Tukey *post hoc* was used to determine statistical significance, where * indicates a *P* ≤ 0.05 and ** ≤0.01. Error bars indicate standard deviation.

H_2_O_2_ production and iron transport are closely linked and that the loss of H_2_O_2_ production is accompanied by activated expression of iron and heme importers (12). Hence, we compared H_2_O_2_ supernatant levels among the isogenic D39 WT, *∆spd_0739*, *∆piuBCDA*, and *∆spd_0739∆piuBCDA* strains during cultivation in THYB (Fig. 5). Inactivation of *spd_0739* resulted in an ~70% decrease in the extracellular levels of H_2_O_2_. The ∆*piuBCDA* strain exhibited a delayed production, but eventually accumulated H_2_O_2_ in the culture medium in equal amounts as the wild-type strain. The phenotype of the double mutant was the same as the single *spd_0739* inactivation. Altogether, these observations establish a strong connection between *spd_0739* expression, iron and heme import, and H_2_O_2_ production in *S. pneumoniae.*

### Externally added nucleoside improve *Δspd_0739* growth at low concentration but become inhibitory in excess in an iron and H_2_O_2_-dependent manner

**Fig 6:**
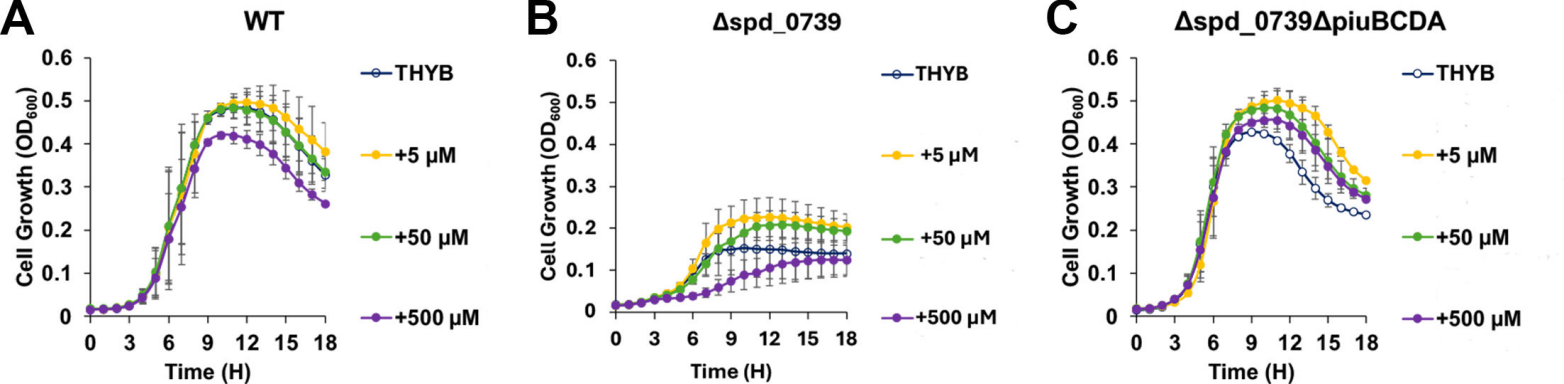
The growth of the ∆*spd_0739* mutant is slightly improved with the addition of nucleoside at low concentration. Cells of the isogenic strains D39 wild type (**A**), Δ*spd_0739* (**B**), and Δ*spd_0739*Δ*piuBCDA* double mutant (**C**) were used to inoculate fresh THYB with increasing concentrations of nucleoside mix (i.e., adenosine, guanosine, cytidine, uridine, and thymidine in equimolar concentration). The experiments (conducted as described in Fig. 2) were done in triplicates and repeated twice. Each curve is a derived triplicate average from representative experiments. Error bars indicate standard deviation.

Inactivation of *spd_0739* (aka *pnrA*) resulted in a reduction of guanosine uptake by pneumococci cultured in RPMI supplemented with 0.5 mM mixture of adenosine, guanosine, cytidine, uridine, and thymidine (31). Interestingly, the presence of 0.5 mM nucleoside mixture resulted in a slower growth of both the D39 wild type and its isogenic *spd_0739* mutant in RPMI. To gain more insights in the possible role of SPD_0739 in nucleoside metabolism and pneumococcal physiology, we examined the growth of the D39 wild type, Δ*spd_0739,* and of the ∆*spd_0739*∆*piuBCDA* double mutant when cultivated in THYB medium supplemented with increasing nucleoside concentration (Fig. 6). The addition of 5 or 50 µM nucleosides did not change the growth of the wild-type strain but had a positive impact on Δ*spd_0739*. Notably, this mutant still exhibited a significant growth attenuation compared with the wild-type strain in THYB. The ∆*spd_0739*∆*piuBCDA* double knockout also benefited from the addition of nucleosides and seems more refractory to their inhibitory impact at high concentration.

**Fig 7:**
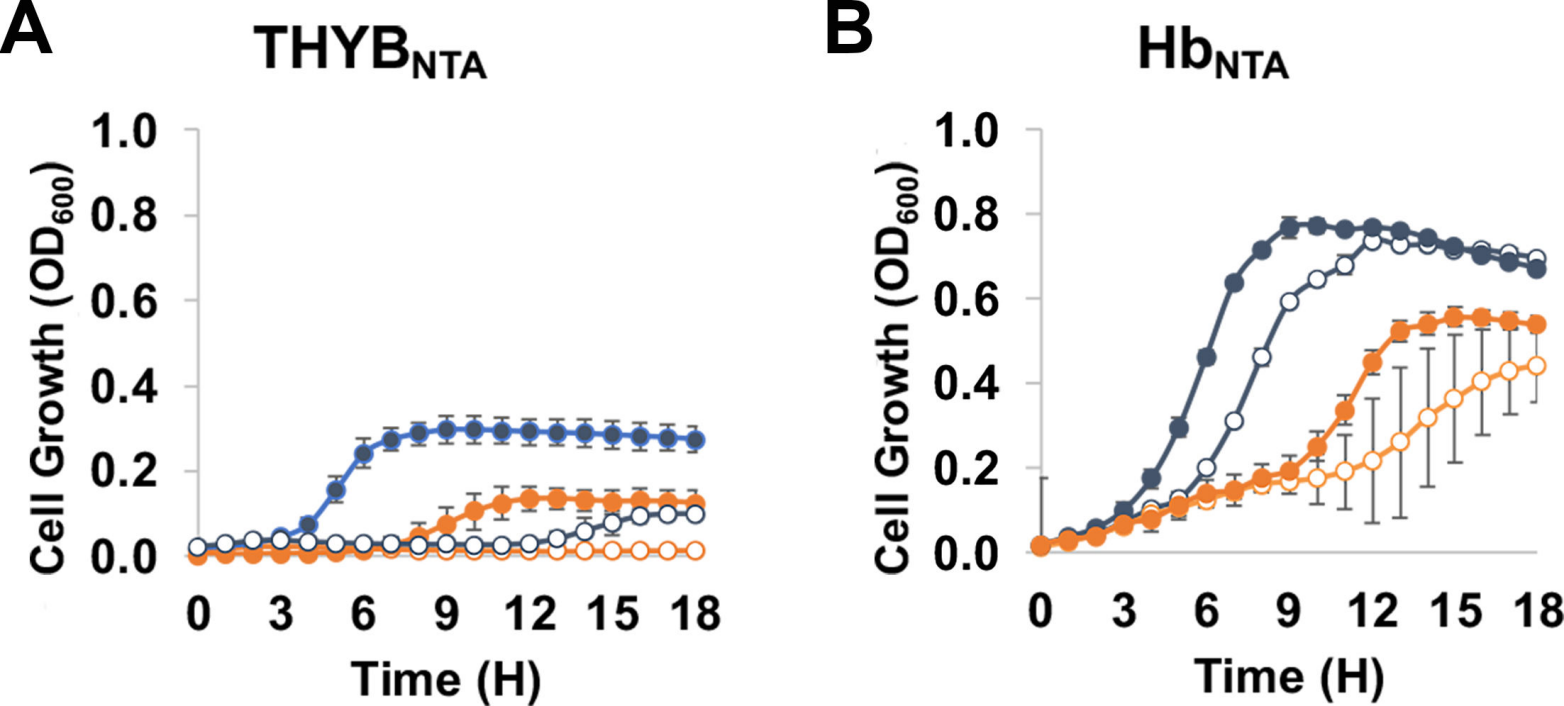
Externally added catalase improves pneumococcal growth in the presence of inhibitory levels of nucleosides. Cells of D39 wild type (**A**) and the Δ*spd_0739* strain (**B**) were grown in THYB, or in THYB supplemented with 0.5 mM nucleoside mix (+Nucleosides), 200 U/µL catalase (+Catalase), or catalase and 0.5 mM nucleosides (+Catalase and Nucleosides). The experiments (conducted as described in Fig. 2) were done in triplicates and repeated twice.Growth is expressed in optical density (OD_600_) as measured every hour for 18 h. All experiments were done in triplicates and repeated twice. Each curve shown is derived from an average of three bioreplicates from representative experiments. Error bars indicate standard deviation.

Ferric-bound nucleosides or nucleotides react with H_2_O_2_ to produce damaging hydroxyl radicals (48), which could be the reason for their negative growth effect in excess. To test this possibility, we included catalase in the growth medium supplemented with 0.5 mM nucleosides. The wild type and the Δ*spd_0739* strain grew better in THYB containing catalase, likely relieving the oxidative stress endogenously produced H_2_O_2_ imposes on pneumococci (Fig. 7). The significant improvement in the Δ*spd_0739* growth in the presence of catalase highlights the high burden oxidative stress has on the physiology of this mutant (Fig. 7B). As expected, the presence of catalase alleviated nucleoside toxicity for both the wild type and the mutant strain. Still, Δ*spd_0739* growth in the presence of nucleosides and catalase was a bit delayed compared to its growth with catalase only.

**Fig 8:**
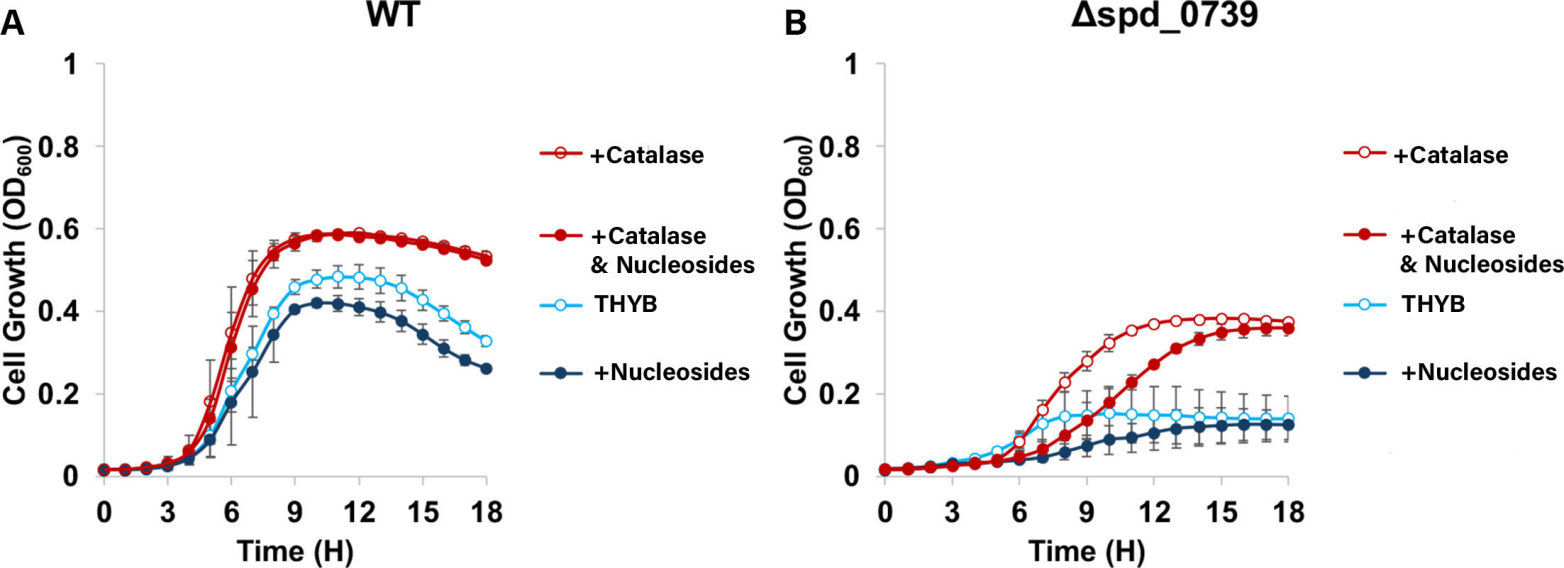
Externally added nucleosides benefit pneumococci growth when the free iron in the medium is chelated**.** Growth of the wild-type D39 (blue) and isogenic mutant *spd_0739* (orange) mutant in THYB with 3 mM NTA (THYB_NTA)_ (**A**) or THYB_NTA_ with 20 µM Hb (**B**)**.** The cultures were allowed to grow without (empty symbols) or with (full symbols) the addition of 0.5 mM nucleoside mixture. The experiments (conducted as described in Fig. 2) were done in triplicates and repeated twice. Growth is expressed in optical density (OD_600_) as measured every hour for 18 h. Each curve shown is derived from an average of three bioreplicates from representative experiments. Error bars indicate standard deviation.

We also tested the growth of the wild type and the Δ*spd_0739* mutant in the iron-depleted medium, THYB_NTA_, in the presence and absence of 0.5 mM nucleosides and hemoglobin. Unlike the negative impact nucleoside excess had on pneumococci growth in THYB, the addition of nucleoside THYB_NTA_ rescued some growth of the wild-type strain, although overall growth was very limited (Fig. 8A). Nucleoside addition also improved growth in THYB_NTA_ supplemented with 20 µM hemoglobin, facilitating higher biomass by both the wild type and the *∆spd_0739* strains. Hence, the chelation of free iron from the medium allows *S. pneumoniae* to enjoy the nutritional benefit of the externally added nucleosides. Still, the ∆*spd_0739* exhibited a significantly longer lag and grew to a lower final OD_600_ compared with the wild type (Fig. 8B).

## DISCUSSION

Ligand capture and delivery across the cytoplasmic membrane is a critical step in nutrient acquisition. ABC transporters carry out this step during the importation of iron or iron complexes in bacteria, delivering their substrate from the periplasm (in Gram-negative bacteria) or the extracellular compartment (in Gram-positive organisms) into the cytoplasm (49, 50). Six ABC transporters have been identified in *S. pneumoniae* that are involved in iron acquisition (using *S. pneumoniae* D39 gene tags): PitADBC (0223–0227) (25, 40), Pit2ABCD (SPD_1607–1609) (44), Pia/FhuABCD (SPD_0915–0918) (40, 46), Piu/FatBCDA (SPD_1649–1652) (11, 36, 40), SPD_0088–0090 (26), and SPD_0739–0742 (9, 30). These transporters are part of the pneumococcal core genome, and several serve as protective antigens whose loss is associated with attenuated virulence (27, 40, 44, 51). There are inconsistencies, however, among independent studies regarding the identity of the cognate ligand for several of the pneumococcal iron transporters. These discrepancies contribute to the lack of clarity regarding the molecular basis of iron homeostasis in this principal human pathogen. This investigation addressed the functional differences reported for the binding protein SPD_0739. The findings are consistent with SPD_0739’s proposed role in nucleoside import and do not support the previous suggestion that it mediates heme uptake. Nevertheless, this investigation identifies SPD_0739 as a new effector linking nucleoside status, H_2_O_2_ production, and iron uptake.

We recently learned that Spbhp-37, a heme- and hemoglobin-binding protein initially identified in the R6 strain (30), is also known as PnrA, which binds nucleosides and is part of a nucleoside ABC importer described in TIGR4 and D39 strains (31, 33, 52). The differences between the studies cannot be attributed to strain differences since Spbhp-37/PnrA proteins are highly conserved among pneumococcal strains, exhibiting 98.3% amino acid identity and 99.7% similarity among R6, D39, and TIGR4. This conservation renders Spbhp-37/PnrA orthologs effectively indistinguishable proteins, likely fulfilling the same function. We first tested heme binding by a recombinant SPD_0739 to address the functional conundrum. The gradual formation of a Soret band we observed upon heme titration confirmed that SPD_0739 binds heme *in vitro* (Fig. 1A). Testing hemoglobin binding by immobilized bacteria showed that an *spd_0739* mutant binds less than the wild-type strain (Fig. 1C), demonstrating SPD_0739’s contribution to pneumococcal interactions with hemoglobin *in vivo*. Further analysis revealed that the growth hindrance exhibited by the Δ*spd_0739* strain in THYB or when cultivated on hemoglobin–iron is relieved with the removal of the iron acquisition system encoded by *piuBCDA* genes (Fig. 2), suggesting that the loss of *spd_0739* does not exacerbate iron shortage. Moreover, knocking out *spd_0739* in either the wild type or the ∆*piuBCDA* background leads to an increase (rather than a decrease) in the cellular heme levels (Fig. 3B) and sensitizes pneumococci to the toxicity of externally added heme (Fig. 3C). We also discovered that the Δ*spd_0739* strain displays an elevated expression of heme and iron transport genes, including *spd_0090* (26), *pitB* (*spd_0225*) (25, 40), and *pitA2* (*spd_ 1609*) (25, 40). Together, these observations strongly argue against the notion that SPD_0739 imports heme; instead, they suggest that SPD_0739 acts to limit heme uptake, although it binds heme and hemoglobin.

It was previously reported that the loss of *spd_0739* results in a decreased guanosine import by pneumococci cultivated in RPMI supplemented with a nucleoside mixture (31). By testing the impact of externally added nucleosides over a concentration range in this study, we found that the Δ*spd_0739* strain grew to a higher density in THYB supplemented with 5 or 50 µM nucleoside mixture (Fig. 6B). The ∆*spd_0739*∆*piuBCDA* double mutant, but not the parent wild-type strain, also benefited from the nucleoside addition (Fig. 6A and B). The observation that *spd_0739* inactivation limits bacterial growth (albeit to a small extent) in a nucleoside-dependent manner agrees with the nucleoside import function. Consistent with this role is *spd_0739* chromosomal location next to the rest of the transporter components (*spd0740-42*) and downstream to the nucleoside salvaging genes, pyrimidine-nucleoside phosphorylase (*pdp*, *spd_0736*), deoxyribose-phosphate aldolase (*deo*C, *spd_0737*), and cytidine deaminase (*cdd-1*, *spd_0738*). Pdp catalyzes the phosphorolytic cleavage of pyrimidines into precursors for nucleic acid synthesis (53); its downregulation in the *spd_0739* knockout strain (Fig. 3B) further links the function of these two genes. Preincubation or coincubation with guanosine did not prevent heme binding to *spd_0739* (Fig. 1B). More studies are required to determine if heme impacts the binding or import of nucleosides by SPD_0739.

Adding 0.5 mM nucleoside mixture to THYB adversely influenced pneumococcal growth (Fig. 6), with the ∆*spd_0739*∆*piuBCDA* double mutant exhibiting the most resistance to the nucleoside’s harmful impact when in surplus. Supplementing RPMI with 0.5 mM nucleoside mixture also results in a slower growth of D39 strains (31). Here, we found that including catalase in the medium (Fig. 7) or chelating free iron with NTA (Fig. 8A and B) relieved the negative influence of nucleoside in high concentration. These observations suggest that nucleoside interactions with ferric iron and hydrogen peroxide and the associated production of reactive oxygen species (48) contribute to the harmful influence of nucleoside excess. Interestingly, the addition of 0.5 mM nucleoside mixture to THYB_NTA_ or THYB_NTA_ with hemoglobin facilitated a notable growth of the wild-type strain in the iron-depleted THYB_NTA_ (Fig. 8A) and improved the growth of both the wild type and the Δ*spd_0739* strains in THYB_NTA_ with hemoglobin. The nucleoside addition likely promotes growth in THYB_NTA_ by alleviating the pressure iron starvation puts on DNA synthesis.

In addition to exhibiting higher iron and heme import gene expression, the ∆*spd_0739* mutant produces less H_2_O_2_ than the wild type (Fig. 5). Pyruvate oxidase (SpxB), which in the presence of oxygen, converts pyruvate to acetyl phosphate while releasing CO_2_, creates the majority of H_2_O_2_ in *S. pneumoniae* (15). The transcriptional regulator, SpxR, activates *spxB* expression in response to adenosyl or CoA-containing compounds (54). Hence, we speculate that the inactivation of *spd_0739* leads to a decrease in the cellular levels of adenosyl nucleosides and indirectly to a reduction in *spxB* activation by SpxR. Interestingly, the absence of *spxB* renders S*. pneumoniae* dependent on the pyruvate dehydrogenase complex (PDHC) activity for acetyl-CoA production (15). PDHC activity requires the iron–sulfur protein ferredoxin. Hence, reduced *spxB* expression can help explain the increased need for iron. The iron importers induced in the ∆*spd_0379* mutant are also activated in a ∆*spxB*∆*lctO* strain, which does not produce any H_2_O_2_ (12). Therefore, it seems likely that the negative impact on H_2_O_2_ production drives the activation of iron transport genes in ∆*spd_0739* by an unknown mechanism. In that context, it is interesting to mention that limited aerobic growth, as used under our experimental setup, induces the expression of several enzymes in the pyruvate node, including PDHC and PflB*,* which depend on iron, as well as enzymes involved in the synthesis of iron–sulfur clusters, and the *piuB* gene (15). Hence, iron is an important cofactor under these growth conditions.

Surprisingly, inactivating the *piuBCDA* transporter relieves most of the growth attenuation exhibited by Δ*spd_0739* (Fig. 2), suggesting that the loss of SPD_0739 renders *piuBCDA* noxious. Early studies suggested that the PiuBCDA proteins import heme and that PiuA, the substrate-binding component, binds heme and hemoglobin (11). Our observations that *piuBCDA* knockout results in reduced hemoglobin binding (Fig. 1B) and a lower heme content ([12] and Fig. 3B) are in agreement with these studies. Nevertheless, PiuA binds ferric-bis-catechol or monocatechol complexes (such as norepinephrine) with an affinity that is 1,000 times higher than heme (36). Moreover, *S. pneumoniae* encodes proteins that facilitate iron removal and the generation of quinones from ferric–catechol complexes. This pathway is repressed by SirR along with the *piuBCDA* genes in response to quinones (39). Hence, PiuBCDA is likely a high-affinity iron–catechol transporter that also imports heme or contributes indirectly to heme uptake.

Both heme and catechols can be harmful; heme is prooxidant that damages many of the cellular constituents, while catechols can spontaneously convert to reactive quinone species and semiquinone radicals. Accordingly, *piuBCDA* expression is subjected to a complex regulatory circuit, which is sensitive to the environmental, metabolic, and energy status of the bacterium. In addition to SifR, the *piu* promoter is also repressed by RitR during oxidative stress and by the pleiotropic repressor, CodY, in a branched-chain amino acid-dependent manner (25, 37, 55–57). A *codY* knockout mutant accumulates suppressor mutations in the *piu* genes (57), indicating that unregulated expression of the Piu system is harmful. Nevertheless, *spd_0739* loss has only a minor impact on the expression of *codY* (1.4-fold) or *piuB* (1.5-fold, Fig. 4A). Hence, we propose that SPD_0739 either inhibits PiuABCD activity (perhaps on the bacterial surface via direct interactions), or SPD_0739 ligands (i.e., nucleosides) are required for pneumococci to metabolize the substrate imported by Piu proteins.

SPD_0739 is a highly expressed lipoprotein and protective antigen that is conserved among pneumococcal serotypes. A deletion mutant in *spd_0739* is delayed in developing pneumonia post intranasal inoculation in a murine model and exhibits reduced fitness in competition experiments (34). While characterized as a promising vaccine target that serves an important function during infection, the exact role of SPD_0739 in pneumococcal physiology is not fully understood. This study suggests that while SPD_0739 binds hemoglobin and heme, it does not mediate heme import. Instead, it indirectly influences iron and heme metabolism, linking nucleosides and iron status in *S. pneumonia*e.

## MATERIALS AND METHODS

### Bacterial growth and media

Frozen stocks of *S. pneumoniae* were preserved in skim milk–tryptone–glucose–glycerin (STGG) as described (58) and stored at −80°C. *S. pneumoniae* cells from STGG stock, were plated on Trypic Soy blood agar plates (BAPs) and incubated at 37°C. Cells collected from BAPs following overnight incubation were used to inoculate fresh medium in a starting OD_600_ 0.05. Pneumococci were grown in Todd–Hewitt both containing 0.5% (wt/vol) yeast extract (THYB), iron-depleted THYB (THYB_NTA_), or iron-depleted THYB supplemented with hemoglobin. THYB_NTA_ was prepared by adding 3 mM nitrilotriacetic acid (NTA), 0.55 mM ZnSO_4_, and 0.55 mM MnCl_2_ to fresh THYB followed by filter sterilization (0.45-μm filters). Hemoglobin stock solutions were prepared fresh by resuspending lyophilized powder of human hemoglobin (Sigma Aldrich) in 1× phosphate-buffered saline (PBS) (1 mM). Hemoglobin was then added at the indicated concentration, and the medium was filter sterilized (0.45 μm filters). Supplementations with nucleosides received a 0.5-mM cocktail of adenosine, guanosine, cytidine, uridine, and thymidine (Sigma-Aldrich). Catalase, 200 U/µL (Sigma-Aldrich), was added when needed. Pneumococci were grown in 96-well flat bottom tissue culture plates at 37°C in a Multiscan Skyhigh spectrophotometer (Thermo Scientific). Optical density was measured hourly after brief shaking.

### Strain and plasmid construction

Construction of the single *∆spd_*0739 and *∆piuBCDA* was generated in previous studies as stated (9, 12). Double ∆*spd_0739*∆*piuBCDA* mutants were accomplished by replacing the *spd_0739* open reading frame (ORF) with the spectinomycin resistance gene, *aad9,* in both the wild type and a *∆piuBCDA::ermC* mutants (12). In these constructs, the *aad9* expression is under *spd_0739* promoter and terminator signals. The mutant allele containing *add9* ORF flanked by the 5′ and 3′ genomic regions of the *spd_0739* gene was prepared using the GeneArt seamless cloning kit (Thermofisher Scientific). Briefly, the appropriate genomic segments were amplified from the D39 chromosome using the primer sets ZE 966 F /ZE 967 R and ZE 970 F/ZE 971 R. The *aad9* gene and the pUC19 vector were amplified from pJRS525 (59) and pUC19-L plasmids using the primer sets ZE 968 F/ZE 969 R and ZE 964 F/ZE 965 R, respectively. All PCR fragments were purified (using the MinElute PCR Purification Kit, Qiagen) and cloned into One Shot TOP10 *E. coli* strain, generating plasmid pEW109. The resulting allele was then amplified (from pEW109) and transformed into competent D39 cells using standard protocols. The mutants were selected on BAPs containing spectinomycin (100 µg/mL). The mutation was confirmed by PCR in the resulting spectinomycin-resistant clones using the primer set ZE 966 F/ZE 971 R.

### Determination of heme content

Fresh THYB_NTA_ containing 20 µM hemoglobin was inoculated with *S. pneumoniae* OD_600_ 0.05. Cultures were grown in 12-well flat-bottom microtiter plates incubated at 37°C. Six-milliliter culture samples (at OD_600_ 1.0) were collected at mid-log as determined by growth kinetics. The cells were harvested, washed three times within PBS, resuspended in 2 mL of dimethylsulfoxide (DMSO), and sonicated (20% amplitude for 30 s). Heme was extracted by acidified chloroform, and its concentration was determined using a standard curve made with hemin solutions in DMSO as described. Briefly, 2 mL of 50 mM glycine buffer, pH 2.0, 0.1 mL of 4 N HCl (pH 2.0), 0.2 mL of 5 M NaCl (pH 2.0), and 2 mL of chloroform: isopropanol was added to the cell lysates. The reactions were mixed vigorously and were allowed to incubate at room temperature for 1 min. The absorbance of the organic phase at 388, 450, and 330 nm was recorded and fed into the correction equation Ac = 2 × A388 − (A450 + A330). Hemoglobin (if present in the culture medium) was removed by filtration before heme extractions.

### Hemoglobin-binding ELISA

Overnight pneumococcal cultures grown with 200 U/µL of catalase (Sigma Aldrich) (5 mL) were harvested, washed with phosphate-buffered saline w/ Tween 20 (5 mL) three times, and used to coat microtiter plates (OD_600_ 1.0 in 50 µL) overnight at 4°C at OD_600_ 1.0. After blocking, cells were allowed to interact with 0–7.5 µM hemoglobin in PBS for 1 h at 37°C. The 1° Anti-Human Hemoglobin antibody (1:8,000) (Sigma) and the 2° AP-Conjugated Anti-Rabbit antibodies (1:10,000) (Sigma) were allowed to interact with the coated wells for 1 h at room temperature (RT), and binding was detected with p-nitrophenyl phosphate substrate solution (ThermoFisher).

### Quantification of hydrogen peroxide concentration in pneumococcal growth media

Pneumococcal cells collected from BAP following overnight growth at 37°C were used to inoculate fresh THYB OD_600_ 0.05 (6 mL in 15-mL Falcon tubes). The supernatant was prepared from culture samples by centrifugation and filtration (0.45 μm. The sample H_2_O_2_ content was measured using the Quantitative Peroxide Assay Kit (ThermoFisher) per the manufacturer’s instructions. A serial dilution of 30% H_2_O_2_ in THYB was used to generate a standard curve, from which we derived the H_2_O_2_ concentration in media samples.

### Purification of His_6_-SPD_0739 proteins

The pEW111 (expressing His_6_-Spd_0739) plasmid was transformed into Invitrogen Chemically Competent BL21(DE3) cells for expression. Overnight cultures of BL21(DE3)/pEW111 from a glycerol stock were diluted in fresh Luria–Bertani (LB) media and grown at 37°C at 225 rpm. The expression of *His_6_-spd_0739* was induced with 0.5 mM isopropyl β-D-1-tiogalactopyranosie (IPTG) at OD_600_ 0.8, and the cultures were incubated overnight at 20°C with 180 rpm. The following day, cells were harvested. Cell pellets were resuspended in 20 mM Tris (pH 8.0), 100 mM NaCl, and 0.1% Triton X-100. One cOmplete EDTA-free Protease Inhibitor Tablet (Roche #1183670001) per 500 mL of grown culture was added before sonification. The cellular debris was pelleted by centrifugation at 20,000 × *g* for 30 min at 4°C, and the lysate was filtered using a 0.45-μm filter unit. Protein was purified on an AKTA FPLC using Cytiva HisTrap HP columns. Imidazole was removed via buffer exchange in Buffer A without imidazole (50 mM potassium phosphate, 250 mM NaCl) using Ultra-15 centricon filters (molecular weight cutoff of 30,000, Amicon). Protein purification and size were confirmed using sodium dodecyl sulfate-polyacrylamide gel electrophoresis (SDS-PAGE). Protein concentration was determined by the ThermoScientific Lowry Protein Assay Kit (23240). The buffer used for reconstitutions and degradations consisted of 20 mM sodium phosphate and 500 mM NaCl (pH 7.4). Arginine (30 mM) and 10% glycerol were added to the buffer for protein storage.

### Heme titration

A stock solution of hemin chloride in DMSO was prepared. The absorbance of a 1:1,000 dilution of the stock solution at 404 nm was recorded, and the concentration of hemin chloride in the stock solution was calculated using Beer’s law (A = εbc, where hemin in DMSO ε_404_ = 188,000 m^−1^ cm^−1^. Protein samples were diluted in His-tag running buffer A (without imidazole) to 10 µM. Absorbance from 250 to 700 nm was recorded before the addition of hemin chloride. Hemin chloride was added to 100-µL aliquots of 10 µM protein to a final hemin chloride concentration of 2 µM, incubated with stirring at RT for 5 min, and the absorbance from 250 to 700 nm was scanned and recorded. This was repeated for hemin chloride concentrations of 2, 4 , 6 and 8, 10, 20, 30, and 40 µM. His-tag buffer A alone was similarly incubated with 2, 4, 6 and 8, 10, 20, 30, and 40 µM of hemin chloride. These heme-containing buffer solutions were scanned as blanks for the UV-visible spectra of the protein solution containing corresponding concentrations of hemin chloride. Guanosine was prepared in Buffer A with 0.05 M glacial acetic acid to help with solubility and diluted into a final concentration of 40 µM in Buffer A with 10 µM of His6x-SPD_0739 prior to addition of heme.

### qRT-PCR analysis

Quantitative RT-PCR (qRT-PCR) analysis was carried out using the Power SYBR Green RNA-to-Ct 1-Step Kit (Applied Biosystems) and StepOne DNA PCR machine (Applied Biosystems) according to the manufacturer’s specifications. A 25-ng RNA was used per qRT-PCR reaction, and each reaction was done in duplicates from two bioreplicates. Primers used for qRT-PCR are listed in Table 1. The relative expression was normalized to the endogenous control *gyrB* gene, and fold changes were calculated using the comparative 2−ΔΔCT method. Iron genes were selected due to associations mentioned in the literature. STRING predictive software was used to predict genes associated with *spd_0739* (60).

**TABLE 1:** Strains, plasmids, and primers

*S. pneumoniae*	Description	Source or references
D39	Avery strain, clinical isolate, WT (capsular serotype 2), CSP1	(61, 62)
D39 *∆spd_0739*	D39 derivative with spd_0739::ermB mutation	(9)
D39 *∆piuBCDA*	D39 derivative with ∆piuBCDA::ermC mutation (erm^R^)	(12)
D39 *∆spd_0739∆piuBCDA*	D39 derivative with spd_0739::aad9 (spec^R^) allele and ∆piuBCDA::ermC mutation (erm^R^)	This study
*E. coli*		
One shot Top10	Cloning strain	Invitrogen
BL21 (DE3)	Expression strain	Invitrogen
Plasmids		
pAF103	pUC19 derivative carrying the ∆piuBCDE::ermC allele. AmpR	(12)
pEW109	pUC19 derivative carrying the ∆spd_0739::aad9 allele pUC19. AmpR	This study
pEW111	pET151 for expression of His_6_-Spd_0739. Amp^R^	This study

Primers			
Target	Primer name	Sequence (5′–3′)	Comments
Puc19L	ZE 730-FW	GTCTGGAAGGCATGCAAGCTTGGCGTAATCAT	Sense primer for cloning of the pUC19 segment of pAF103
pUC19L	ZE 731-RV	GAACATGAGTACCGAGCTCGAATTCACTGGCC	Antisense primer for cloning of the pUC19 segment of pAF103
5′piuB & upstream region (D39)	ZE 732 FW	CTCGGTACTCATGTTCTCTTCGACTGCTTCTC	Sense primer for cloning of the left arm of the piu segment of pAF103
5′piuB & upstream region (D39)	ZE 733 RV	CACACGGTGGTAATAGTCTGCATGAGAAGGCC	Antisense primer for cloning of the left arm piu segment of pAF103
*ermC* (pJRS233)	ZE 734-FW	CTATTACCACCGTGTGCTCTACGACCAAAACT	Sense primer for cloning of the ermC segment of pAF103
*ermC* (pJSR233)	ZE 735-RV	TTGCTTGCCAAAGCTGCCGACAACACGGGAGC	Antisense primer for cloning of the ermC segment of pAF103
3′ *piuA* and downstream region (D39)	ZE 736- FW	CAGCTTTGGCAAGCAAGGATGACTACTGGACT	Sense primer for cloning of the right arm of the piu segment of pAF103
3′ *piuA* and downstream region (D39)	ZE 737-RV	TGCATGCCTTCCAGACCACTTTTCCCTTAAAC	Antisense primer for cloning of the right arm piu segment of pAF103
Puc19L	ZE 964-FW	ATCAAAGAGGCATGCAAGCTTGGCGTAATCAT	Sense primer for cloning of the pUC19 segment of pEW109
Puc19R	ZE 965-RV	GTTCAACCGTACCGAGCTCGAATTCACTGGCC	Antisense primer for cloning of the pUC19 segment of pEW109
5′ SPD_0739 upstream region (D39)	ZE 966-FW	CTCGGTACGGTTGAACATGCTAAAAAAGGACT	Sense primer for cloning of the left arm of spd_0739 segment of pEW109
5′ SPD_0739 upstream region (D39	ZE 967-RV	ATCCTCCTCATTACTGAACCTCCTAAATAAGA	Antisense primer for cloning of the left arm of spd_0739 segment of pEW109
Spec cassette (pJRS525)	ZE 968-FW	CAGTAATGAGGAGGATATATTTGAATACATAC	Sense primer for cloning of the spec segment of pEW109
Spec cassette (pJRS525)	ZE 969-RV	TCCATCCATTATAATTTTTTTAATCTGTTATT	Antisense primer for cloning of the spec segment of pEW109
3′ SPD_0739 downstream region (D39)	ZE 970-FW	AATTATAATGGATGGAAGTCATTTCTTAGGAA	Sense primer for cloning of the right arm of spd_0739 segment of pEW109
3′ SPD_0739 downstream region (D39)	ZE 971-RV	TGCATGCCTCTTTGATAGACAAAACCACTTCT	Antisense primer for cloning of the right arm of spd_0739 segment of pEW109
5′ SPD_0739 upstream region (D39) without signal peptide	ZE 962-FW	CACCGGTAACCGCTCTTCT	Sense primer for cloning of spd_0739 in pET151 backbone (pEW111)
3′ SPD_0739 upstream region (D39)	ZE 963-RV	TTATTTTTCAGGAACTTTTACGC	Antisense primer for cloning of spd_0739 in pET151 backbone (pEW111)
gyrB (D39)	ZE 661-FW	GGCACTGTATGGTATCACACAAG	qRT-PCR
gyrB RV	ZE 662	TCTCTAAATTGGGAGCGAATGTC	qRT-PCR
piuB FW	ZE 864	TGATTTCGACCAGCAGACCTG	qRT-PCR
piuB RV	ZE 865	CTGTACTCGGTGCAGCAAACTG	qRT-PCR
PitA2 (Spd_1609) FW	ZE 149-FW	ATTGCCCGTCCTGTACCACC	qRT-PCR
PitA2 (Spd_1609) RV	ZE 150-RV	TGCTGCTTGCTCTGGAGGTT	qRT-PCR
piaA FW	ZE 151-FW	AAAAATGGTGCCGTTGCTGT	qRT-PCR
piaA RV	ZE 152-RV	AAAGTGGTGTTGGAGTGCATGA	qRT-PCR
spd_0225 (pitB) FW	ZE 153-FW	CCAAGCGAAGTTTGGCCTCC	qRT-PCR
spd_0225 (pitB)	ZE 154-RV	CACCTTGGCATGCGCCTTAG	qRT-PCR
spd_0310 FW	ZE 155-FW	TGATCAAGGCAGCGGCTGTA	qRT-PCR
spd_0310 RV	ZE 156-RV	GAAACTGGTGGACCAGCCCT	qRT-PCR
spd_0090 FW	ZE 171-FW	ACGGTCCAGAAGGCAAGAACT	qRT-PCR
spd_0090 RV	ZE 172-RV	ACCAGTGTTCCATCCACCCA	qRT-PCR
codY FW	ZE 167-FW	CCGCCATGGTGACAGCAGTA	qRT-PCR
codY RV	ZE 168-RV	ATCGGGGATTCGCCTTGGTT	qRT-PCR
pdp FW	ZE 281-FW	CTACTGGTGGTGTCGGCGAT	qRT-PCR
pdp RV	ZE 282-RV	GCCAAGACCACGACCACTCA	qRT-PCR
deoC FW	ZE 279-FW	GAGTCAAGGCTGCTGGTGGA	qRT-PCR
deoC RV	ZE 280-RV	AGCTGACGTTCCGATACGGG	qRT-PCR
pnrB FW	ZE 283-FW	ACCGTGGTGTTCCAGCTGTT	qRT-PCR
pnrB RV	ZE 284-RV	ACGTGGGTGCAAGCCTACAA	qRT-PCR
guaA FW	ZE 285-FW	GCAAGTCCTGGTCCTGGGAA	qRT-PCR
guaA RV	ZE 285-RV	ACCACAACGTGGGTGGTCTT	qRT-PCR
